# The Role of Hydrogen on the Behavior of Intergranular Cracks in Bicrystalline α-Fe Nanowires

**DOI:** 10.3390/nano11020294

**Published:** 2021-01-23

**Authors:** Jiaqing Li, Cheng Lu, Long Wang, Linqing Pei, Ajit Godbole, Guillaume Michal

**Affiliations:** 1 School of Mechanical, Materials, Mechatronic and Biomedical Engineering, University of Wollongong, Wollongong, NSW 2522, Australia; jl901@uowmail.edu.au (J.L.); lw010@uowmail.edu.au (L.W.); agodbole@uow.edu.au (A.G.); gmichal@uow.edu.au (G.M.); 2 School of Chemistry, University of Sydney, Sydney, NSW 2006, Australia; lp115@uowmail.edu.au

**Keywords:** hydrogen embrittlement, bicrystalline nanowires, brittle cleavage, ductile emission, cyclic loading

## Abstract

Hydrogen embrittlement (HE) has been extensively studied in bulk materials. However, little is known about the role of H on the plastic deformation and fracture mechanisms of nanoscale materials such as nanowires. In this study, molecular dynamics simulations are employed to study the influence of H segregation on the behavior of intergranular cracks in bicrystalline α-Fe nanowires. The results demonstrate that segregated H atoms have weak embrittling effects on the predicted ductile cracks along the GBs, but favor the cleavage process of intergranular cracks in the theoretically brittle directions. Furthermore, it is revealed that cyclic loading can promote the H accumulation into the GB region ahead of the crack tip and overcome crack trapping, thus inducing a ductile-to-brittle transformation. This information will deepen our understanding on the experimentally-observed H-assisted brittle cleavage failure and have implications for designing new nanocrystalline materials with high resistance to HE.

## 1. Introduction

It has been known for about a century that hydrogen contamination causes severe degradation in the mechanical properties of metals [1,2,3,4,5,6,7]. This phenomenon is generally termed as “hydrogen embrittlement” (HE). With the fast development of high-strength steels and increasing utilization of H energy, HE is becoming an important industrial challenge. For example, high-strength steels are the preferred material in the automotive industry to increase fuel efficiency. When exposed to an aggressive environment, these steels endure the threat of HE [8]. In addition, the HE of steels is also becoming a severe challenge in the design and use of H-pressurized pipes and storage containers [2,7]. Due to the multifaceted nature, a number of HE theories have been reported, including H-enhanced decohesion (HEDE) [9,10,11,12], H-enhanced localized plasticity (HELP) [13,14,15], H-enhanced strain-induced vacancy (HESIV) [16,17], adsorption-induced dislocation-emission (AIDE) [18,19], and defactant concept [20,21]. Notwithstanding intensive studies using progressive experimental techniques, theoretical modelling and simulations [2,15,22,23,24,25,26], there are still strong debates and polarization of opinions on the underlying HE mechanisms [18,19,27,28,29,30].

One important feature of HE phenomenon is brittle fracture. When considering that such a fracture process happens via the crack propagation, it is suppressed if the dislocation emission dissipates energy and blunts the crack tip [31,32]. It seems to be vital to address the issue associated with the inherent competition relationship between the brittle cleavage and ductile emission at a crack tip. If H segregation can decrease the fracture energy to a threshold where the critical stress intensity factor for cleavage $K_{Ic}$ falls below that for dislocation emission $K_{Ie}$, it is possible for the brittle cleavage to occur. Previously, there have been atomistic investigations over H effects on the crack tip behavior [28,29,33,34,35,36,37,38,39]. For example, Song and Curtin [28] proposed that H accumulation at a sharp crack tip in bulk Fe resulted in a high local H concentration such that the dislocation emission could be inhibited, ultimately leaving cleavage as the only deformation mechanism of the crack tip.

Most of the existing studies have focused on H-induced cracking in bulk materials. However, little is known about the influence of solute H on the fracture mechanisms of nanocrystalline materials with a large volume fraction of grain boundaries (GBs). It has been shown that GBs play a critical role in H segregation and failure patterns [40]. Furthermore, “special” GBs such as nanotwins are often utilized by GB engineering to enhance resistance to intergranular HE in metallic systems [41,42]. For example, Fang et al. [41] developed a nanotwinned 304 austenite stainless steel through the dynamic plastic deformation (DPD) treatment, and found that the DPD-annealed sample with 41% nanotwins exhibited a remarkably high HE resistance. Therefore, a deeper understanding of the intergranular crack behavior in nanocrystalline materials during the HE phenomenon is of significance to the improved failure prediction and to the design of HE-resistant nanostructures.

Herein, based on the technique of molecular dynamics (MD) simulations, we use bicrystalline nanowires with symmetric tilt GBs as a platform to explore the H-modified behavior of intergranular cracks at the nanoscale. As steels are more susceptible to HE, α-Fe is selected as the model material. By performing simulations under various loading spectra, we report that the H segregation creates no ultimate cleavage for the predicted ductile cracks along the studied GBs in bicrystalline α-Fe nanowires under monotonic loading, whereas a ductile-to-brittle transition occurs under cyclic loading due to the enhanced H accumulation and suppressed crack trapping in the GB.

## 2. Simulation Methodology

**Figure 1:**
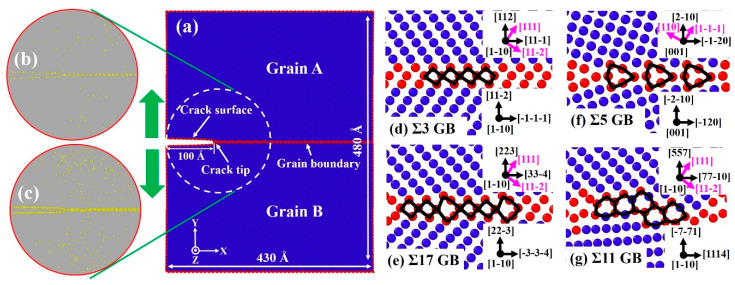
Molecular dynamics (MD) simulation setup for the mode-I fracture along the tilt boundary. (**a**) Geometry and crystallographic orientations of bicrystalline nanowire. (**b**,**c**) H distribution around the crack tip with 45 and 135 mppm H atoms. (**d**–**g**) Atomic configurations of the equilibrium structure of various grain boundary (GB) types. Images are colored by common neighbor analysis (CNA), where atoms with a body-centered cubic (bcc) structure are colored in blue, atoms at the GB and free surface are colored in red, and H atoms are assigned in yellow. The structural units of each GB are outlined by the black line. Possible dislocation slip systems are indicated with pink arrows.

All MD simulations were conducted using the large-scale atomic/molecular massively parallel simulator (Sandia National Laboratories, Albuquerque, NM, USA [43] with the Finnis–Sinclair-type embedded-atom method potential for Fe-H [28]. Four bicrystalline nanowires with tilt boundaries were established according to the specified crystallographic orientations: Σ3 (1 1 2) [1 $\bar{1}$ 0] GB, Σ17 (2 2 3) [1 $\bar{1}$ 0] GB, Σ5 (2 $\bar{1}$ 0) [0 0 1] GB, and Σ11 (5 5 7)_A_($\bar{7}$ $\bar{7}$ 1)_B_ [1 $\bar{1}$ 0] GB, as shown in Figure 1d–g. The simulation domains have dimensions $L_{x}\times L_{y}\times L_{z}$ of about 430 × 480 × 200 Å and a total number of atoms of about 3.6 × 10^6^. A crack of length of 100 Å was created by removing three atomic planes along the boundary, and the periodic boundary condition was prescribed in the *Y* axis to obtain bicrystalline nanowires. An incremental displacement was applied along the *Y* axis for mode I loading according to the anisotropic elastic stress intensity field $K_{I}$ [44]. To investigate the effects of H, H atoms were randomly inserted into simulation models with four H concentrations of approximately 45, 90, 135, and 180 mass parts per million (mppm), respectively. The system was initially loaded to $K_{Iapp}=0.6\;\mathrm{MPa}\;\sqrt{m}$ to drive the H segregation at the crack tip, where $K_{Iapp}$ represents the applied stress intensity factor. Prior to the fracture simulations, the created samples were first equilibrated at a temperature of 700 K for 1 ns, then cooled down to 300 K for 1 ns, followed by further equilibration at 300 K for 3 ns. As shown in Figure 1b,c, H atoms preferentially segregate onto the GB and crack surfaces due to the strong H trapping effect of defects (GBs/cracks) and high diffusion of H atoms in the α-Fe lattice. After obtaining equilibrated Fe-H configurations, the crack was loaded further by imposing successive increments of $\Delta K_{Iapp}=0.001\;\mathrm{MPa}\;\sqrt{m}$ every 1 × 10^−3^ ns. For the cyclic loading cases, loading spectra are presented in Section 3.3. MD simulations were performed under the canonical ensemble (NVT) [45] with time increments of 0.5 and 1 fs for models with and without H, respectively. The Nose-Hoover method [46,47] was used to keep the system temperature at 300 K. Illustration of the simulation results and supplementary movies was achieved by calculating the common neighbor analysis (CNA) parameter at each snapshot in Ovito [48].

## 3. Results

### 3.1. Theoretical Model for Embrittlement

**Figure 2:**
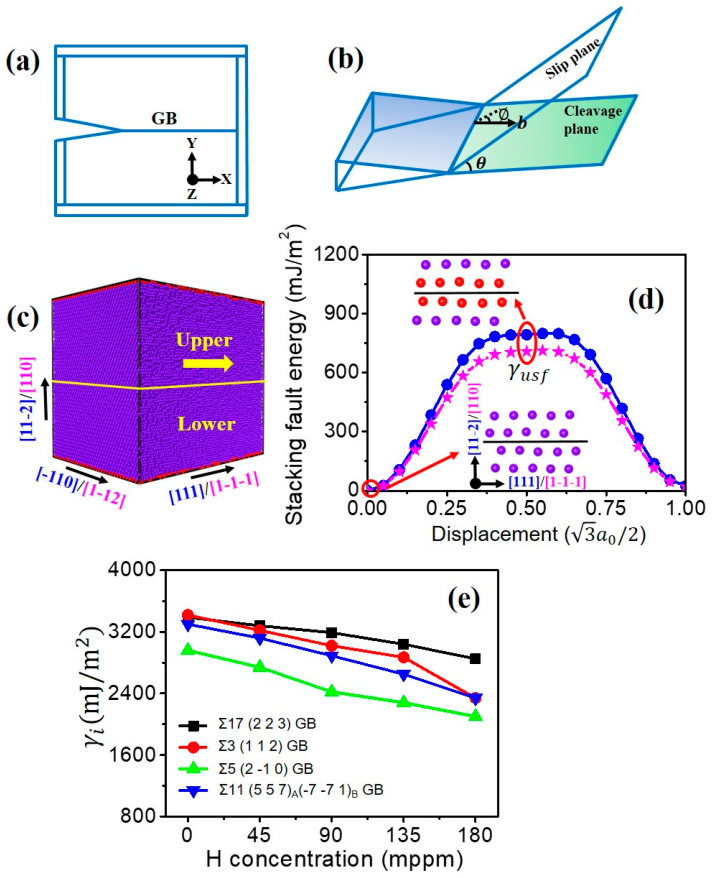
Schematic representations of (**a**) crack along a tilt GB and (**b**) dislocation emission on the slip plane emanating from the crack tip. (**c**) Simulation cell for calculating the generalized stacking fault in $\left(1\;1\;\bar{2}\right)\left[1\;1\;1\right]$ and $\left(1\;1\;0\right)\left[1\;\bar{1}\;\bar{1}\right]$ slip systems. Atoms with the bcc structure are colored by dark blue, and the red atoms stand for the free surface and the stacking fault. (**d**) The generalized stacking fault energy versus fractional displacement in $\left(1\;1\;\bar{2}\right)\left[1\;1\;1\right]$ slip system (blue circle) and $\left(1\;1\;0\right)\left[1\;\bar{1}\;\bar{1}\right]$ slip system (pink star). (**e**) Fracture energy of various GBs versus the H concentration.

As illustrated in Figure 2a, an intergranular crack is inserted along a tilt GB and propagates to the right. According to Griffith’s theory, the critical stress intensity factor for brittle cleavage propagation under mode Ι loading is derived as:

(1) $$K_{Ic}=\sqrt{\gamma_{i}/A_{1}}$$

where $\gamma_{i}$ is the fracture energy, and $A_{1}$ is a constant depending on the anisotropic linear elasticity $c_{ij}$ [49,50]:

(2) $$A_{1}={\left[\frac{\sqrt{c_{22}}}{2}\sqrt{2c_{11}+2c_{12}+c_{44}}\right]}^{-1}$$

The value of $\gamma_{i}$ as a function of the GB type and H concentration is determined by rigidly separating the two grains across a specified plane parallel to the GB and calculating the difference between the initial and final system energies divided by the GB area. See more calculation details in our previous study [12,51]. The corresponding results in Figure 2e clearly show that for all investigated GBs, $\gamma_{i}$ is decreased with the increasing H concentration.

**Figure 3:**
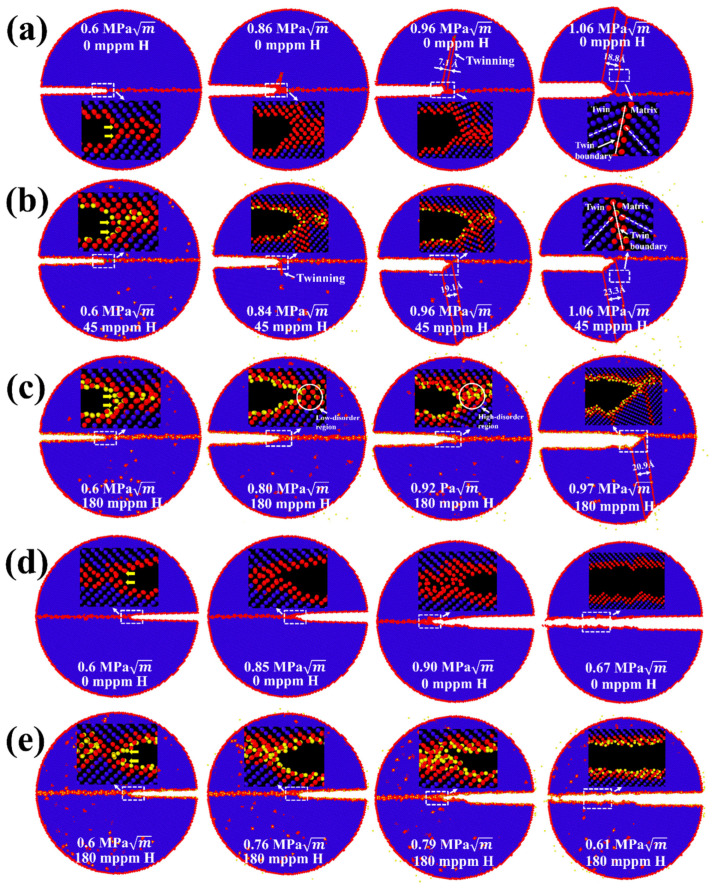

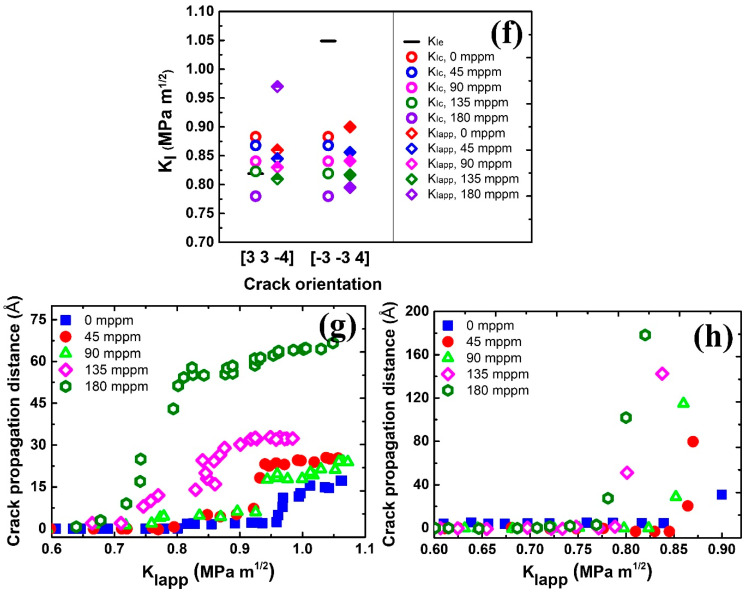
MD snapshots illustrating the crack propagation in the simulations of Σ17 (2 2 3) GB along (**a**) [$3\;3\;\bar{4}$] direction with 0 mppm H atoms, (**b**) [$3\;3\;\bar{4}$] direction with 45 mppm H atoms, (**c**) [$3\;3\;\bar{4}$] direction with 180 mppm H atoms, (**d**) [$\bar{3}\;\bar{3}\;4$] direction with 0 mppm H atoms, and (**e**) [$\bar{3}\;\bar{3}\;4$] direction with 180 mppm H atoms. (**f**) Overview of critical stress intensity factors $K_{Ie}$ and $K_{Ic}$, and the global applied stress intensity factor $K_{Iapp}$ in dependence on crack orientation and H concentration. The partially filled diamonds indicate ductile emission, while solid diamonds indicate brittle cleavage. Crack propagation distance versus the stress intensity $K_{Iapp}$ with various H concentrations along (**g**) [$3\;3\;\bar{4}$] direction and (**h**) [$\bar{3}\;\bar{3}\;4$] direction.

**Figure 4:**
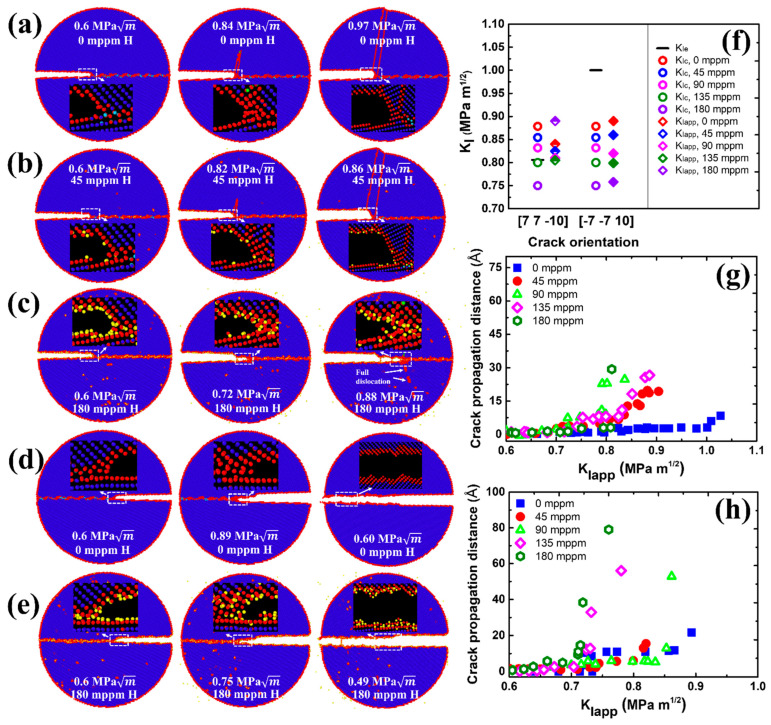
MD snapshots illustrating the crack propagation in the simulations of Σ11 (5 5 7)_A_($\bar{7}$ $\bar{7}$ 1)_B_ GB along (**a**) [$7\;7\;\bar{10}$] direction with 0 mppm H atoms, (**b**) [$7\;7\;\bar{10}$] direction with 45 mppm H atoms, (**c**) [$7\;7\;\bar{10}$] direction with 180 mppm H atoms, (**d**) [$\bar{7}\;\bar{7}\;10$] direction with 0 mppm H atoms, and (**e**) [$\bar{7}\;\bar{7}\;10$] direction with 180 mppm H atoms. (**f**) Overview of critical stress intensity factors $K_{Ie}$ and $K_{Ic}$, and the global applied stress intensity factor $K_{Iapp}$ in dependence on crack orientation and H concentration. The partially filled diamonds indicate ductile emission, while solid diamonds indicate the brittle cleavage. Crack propagation distance versus the stress intensity $K_{Iapp}$. with various H concentrations along (**g**) [$7\;7\;\bar{10}$] direction and (**h**) [$\bar{7}\;\bar{7}\;10$] direction.

Here, Rice’s model of dislocation emission at a crack tip is adopted to study the intrinsic ductility of the GB [32]. In this model, the critical stress intensity factor required for the dislocation emission is defined as:

(3) $$K_{Ie}=\frac{1}{\cos^{2}\left(\theta /2\right)\sin\left(\theta /2\right)}\sqrt{\frac{2G}{1-v}\gamma_{usf}\left[1+\left(1-v\right)\tan^{2}\emptyset \right]}$$

where G is the shear modulus, v is the Poisson ratio, $\gamma_{usf}$ is the unstable stacking fault energy, θ and ∅ are the angle between the cleavage and slip planes, and the angle between the crack normal and the Burgers vector, respectively (see Figure 2b). For calculating $\gamma_{usf}$ using MD, a single crystal is created and shown in Figure 2c. The stacking fault energy curves are determined by rigidly displacing the upper slab on $\left(1\;1\;\bar{2}\right)/\left(1\;1\;0\right)$ plane along $\left[1\;1\;1]/[1\;\bar{1}\;\bar{1}\right]$ direction, while fixing the lower slab and calculating the energy change in the whole simulation cell. The fractional displacement is $0.05\;\times \;\sqrt{3}a_{0}/2$ each step, where $a_{0}$ is the lattice parameter and $\sqrt{3}a_{0}/2$ is the magnitude of Burgers vector of full dislocation. The value of $\gamma_{usf}$ is obtained when the fractional displacement reaches half of the magnitude of Burgers vector of full dislocation, as shown in Figure 2d. In the present study, H atoms mainly occupy at the crack tip and GB, while few H atoms exist at the slip plane. Further, some studies have revealed that the H segregation into the slip plane marginally changes the $\gamma_{usf}$ [50,52]. Therefore, the value of $\gamma_{usf}$ for pure Fe is used in all cases. It needs to be mentioned that directional anisotropy exists for intergranular crack propagation. With different values of θ and ∅, the ductile behavior (dislocation emission) may be expected for one crack tip ($K_{Ie}<K_{Ic}$), while the brittle behavior (cleavage) may occur for the opposite crack tip within the GB plane ($K_{Ic}<K_{Ie}$). For example, the crack of Σ17 (2 2 3) GB exhibits the ductile behavior along [$3\;3\;\bar{4}$] direction, while showing the brittle fracture along [$\bar{3}\;\bar{3}\;4$] direction (Figure 3). Likewise, in the case of Σ11 (5 5 7)_A_($\bar{7}$ $\bar{7}$ 1)_B_ GB, $\left[7\;7\;\bar{10}\right]$ is the ductile direction, while $\left[\bar{7}\;\bar{7}\;10\right]$ is the theoretically brittle direction (Figure 4).

### 3.2. Dislocation Emission and Cleavage of Crack Tip under Monotonic Loading

Figure 3a–e shows atomic configurations of the intergranular crack along the Σ17 (2 2 3) GB for two cracking directions. For crack propagation along the [$3\;3\;\bar{4}$] direction (Figure 3a), the crack tip plasticity takes place by emission of twins at $K_{Iapp}=0.86\;\mathrm{MPa}\;\sqrt{m}$ in the absence of H. This behavior is in accordance with Rice’s model ($K_{Ie}<K_{Ic}$ in Figure 3f). Upon increasing the applied load, the nucleated twins propagate as the twinning space widens from 7.1 to 18.8 Å, further blunting the crack tip. Under an H environment (45 mppm H atoms), the ductile emission behavior is similar, but Figure 3b shows that the crack tip plasticity is triggered at the opposite grain and at a lower applied load $K_{Iapp}=0.84\;\mathrm{MPa}\;\sqrt{m}$. This can be ascribed to the H segregation that disrupts the atomic structure around the crack tip (compare insets in Figure 3a,b at $0.6\;\mathrm{MPa}\;\sqrt{m}$). The disordered structure obviously serves as a plasticity source [53], promoting the slip activity from the crack tip. As the H concentration increases, the fracture energy decreases (Figure 2e). Therefore, the corresponding $K_{Ic}$ is reduced according to Griffith’s theory, as indicated in Equation (1). For the model with a high H concentration (180 mppm), the calculated $K_{Ic}$ drops to $0.78\;\mathrm{MPa}\;\sqrt{m}$, less than $K_{Ie}=0.82\;\mathrm{MPa}\;\sqrt{m}$ (Figure 3f), suggesting that a ductile-to-brittle shift at the crack tip will occur. However, the simulation results demonstrate that the behavior of the propagating crack is more complex than a simple cleavage process (Figure 3c). At a load of $0.80\;\mathrm{MPa}\;\sqrt{m}$, the crack is seen to cleave along the boundary plane without any ductile emission, whereas such a brittle cleavage stops at a higher load, in which the emission of twins and blunting of crack tip resumes. The process of twinning propagation continues with the applied load; the crack tip thus exhibits the ductile behavior at an H concentration of 180 mppm. This phenomenon may be correlated with crack trapping in GBs. The crack advances through the region of low-disorder atomic structure (Figure 3c at $0.80\;\mathrm{MPa}\;\sqrt{m}$) but is eventually arrested by the high-disorder region (Figure 3c at $0.92\;\mathrm{MPa}\;\sqrt{m}$). Such trapping leads to a higher $K_{Iapp}$, and local plasticity can be triggered in place of the cleavage if $K_{Iapp}>K_{Ie}$ (Figure 3c at $0.97\;\mathrm{MPa}\;\sqrt{m}$). In contrast to the [$3\;3\;\bar{4}$] cracking direction, [$\bar{3}\;\bar{3}\;4$] is the theoretically brittle orientation (Figure 3f). In pure Fe, the fracture along the boundary plane occurs in a perfectly brittle manner at $0.90\;\mathrm{MPa}\;\sqrt{m}$ (Figure 3d), which is approaching the predicted value of $0.88\;\mathrm{MPa}\;\sqrt{m}$. The presence of H causes a reduction in $K_{Ic}$, and thus the $K_{Iapp}$. For example, it is seen from Figure 3e that with 180 mppm H the applied load is decreased to $0.79\;\mathrm{MPa}\;\sqrt{m}$ for the onset of cleavage. The results in Figure 3h further show that the crack propagation distance along the H-charged GB is much larger than that along the pure GB at the same $K_{Iapp}$, being indicative of that the H segregation facilitates the cleavage process of the GB crack in the predicted brittle direction.

**Figure 5:**
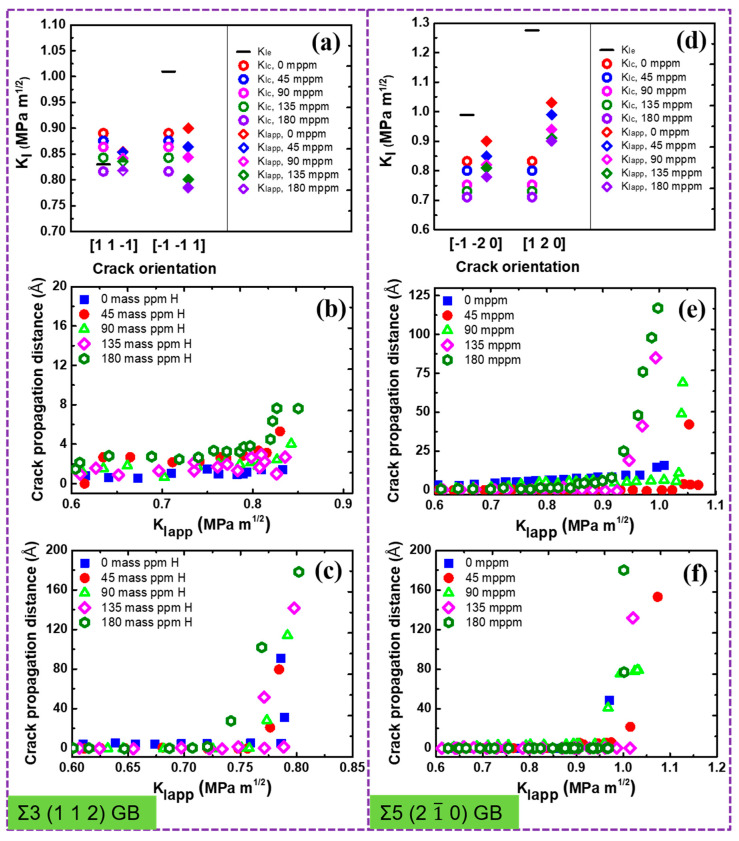
(**a**,**d**) Overview of critical stress intensity factors $K_{Ie}$ and $K_{Ic}$, and the global applied stress intensity factor $K_{Iapp}$ in dependence on the crack orientation and H concentration. The partially filled diamonds indicate ductile emission, while solid diamonds indicate brittle cleavage. Crack propagation distance versus the stress intensity $K_{Iapp}$ with various H concentrations along (**b**) [$1\;1\;\bar{1}$] direction, (**c**) [$\bar{1}\;\bar{1}\;1$] direction, (**e**) [$\bar{1}\;\bar{2}\;0$] direction, and (**f**) [1 2 0] direction.

The crack tip behavior for Σ11 (5 5 7)_A_($\bar{7}$ $\bar{7}$ 1)_B_ GB is qualitatively similar to that for the Σ17 (2 2 3) GB, as seen in Figure 4. The emission of twins with Burgers vector [1 1 1]/6 dominates the plastic deformation of the crack along $\left[7\;7\;\bar{10}\right]$ the ductile direction in the absence of H (Figure 4a). The introduction of H atoms enhances the local plasticity, just as in Σ17 (2 2 3) GB. With the increasing H concentration, the crack tip still exhibits the ductile behavior and no ductile-to-brittle transformation can be observed even though the predicted $K_{Ic}$ is below $K_{Ie}$ (Figure 4c,f). It is interesting to note that rather than the twinning emission, an array of full dislocations with Burgers vector $\left[\bar{1}\;\bar{1}\;1\right]/2$ nucleate at $0.88\;\mathrm{MPa}\;\sqrt{m}$ after a partial cleavage, and then slip away from the crack tip. For the predicted brittle direction, the cleavage advances along the boundary upon continuous loading, and H atoms make it easier for the crack growth (Figure 4d,e,h). Similar results are also observed at Σ3 (1 1 2) and Σ5 (2 $\bar{1}$ 0) GBs, as shown in Figure 5 and Videos S1–S8.

### 3.3. Dislocation Emission and Cleavage of Crack Tip under Cyclic Loading

**Figure 6:**
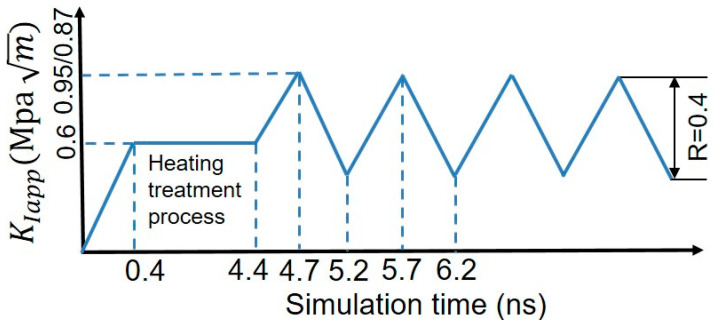
Cyclic loading spectra in the simulations.

**Figure 7:**
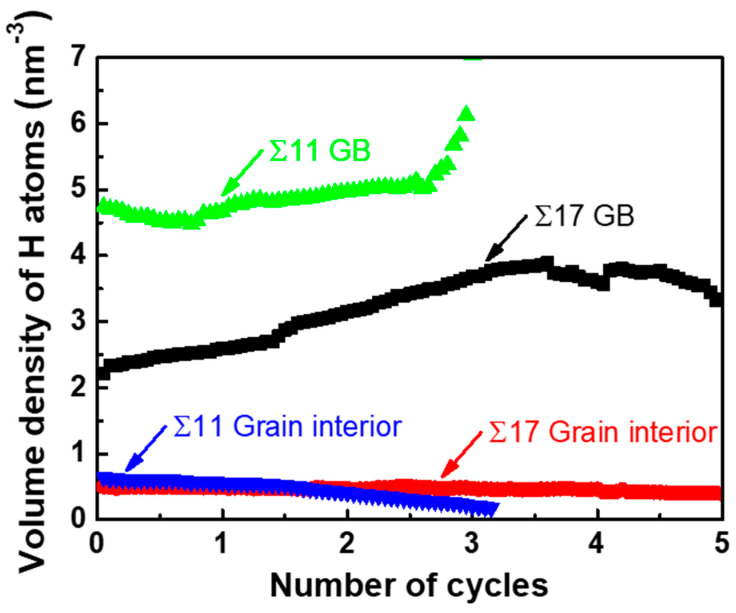
The volume density of segregated H atoms into the GB and the grain interior region versus the number of cycles.

Recent experiments in a near-neutral pH stress corrosion cracking environment have shown that H segregation significantly decreases the threshold stress intensity factor in fatigue tests, which facilitates the occurrence of the brittle fracture [54,55,56,57]. Inspired by the experimental findings, we study the influence of various loading spectra on the crack tip deformation along GBs. Here, only theoretically ductile directions of Σ17 (2 2 3) [1 $\bar{1}$ 0] GB and Σ11 (5 5 7)_A_($\bar{7}$ $\bar{7}$ 1)_B_ [1 $\bar{1}$ 0] GB are considered. The cyclic loading spectra are shown in Figure 6. The maximum value of the applied stress intensity factor is $0.95\;\mathrm{MPa}\;\sqrt{m}$ for Σ17 (2 2 3) GB, and $0.87\;\mathrm{MPa}\;\sqrt{m}$ for Σ11 (5 5 7)_A_($\bar{7}$ $\bar{7}$ 1)_B_ GB, and R ($K_{Iapp,\;min}/K_{Iapp,\;max}$) is 0.4. Figure 7 shows the volume density of segregated H atoms in the GB region and the grain interior for bicrystalline nanowires as a function of the number of cycles. In order to calculate the volume density of segregated H atoms, a slab extending ±1.3 nm perpendicular to the boundary plane was defined as the GB region, and a slab with the 8.0 nm thickness nearby the grain center (grain A/B) was considered as the region of grain interior.

**Figure 8:**
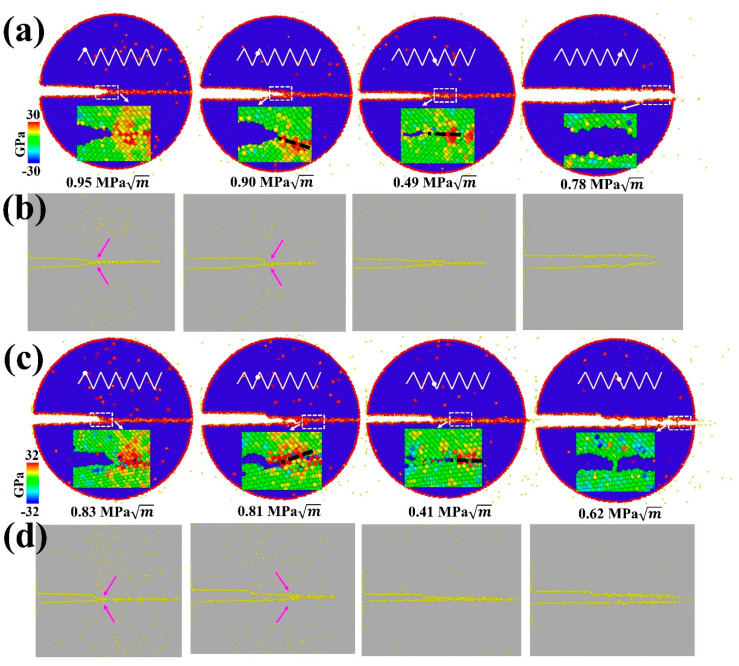
MD snapshots illustrating the crack propagation along the predicted ductile direction of (**a**) Σ17 (2 2 3) GB and (**c**) Σ11 (5 5 7)_A_($\bar{7}$ $\bar{7}$ 1)_B_ GB with 135 mppm H atoms under cyclic loading. The upper inserts are loading spectra, and lower inserts of each image represent the stress field distribution and are colored by the hydrostatic stress value. (**b**,**d**) The corresponding H distribution maps under different stress intensities. The possible cracking path is marked by a dark dash, and H diffusion is indicated with pink arrows.

In contrast to monotonic loading cases, the ductile-to-brittle transition for GB cracks can be observed at Σ17 (2 2 3) GB and Σ11 (5 5 7)_A_($\bar{7}$ $\bar{7}$ 1)_B_ GB under cyclic loading (Figure 8). Specifically, Figure 8a shows that a partial cleavage advances at $0.90\;\mathrm{MPa}\;\sqrt{m}$, which corresponds to the second cycle of the loading phase. Continued cyclic loading leads to the successive separation of the GB structure, with the final rupture occurring in the fifth cycle. As with Σ17 (2 2 3) GB, the crack propagates by cleavage along the predicted ductile direction of Σ11 (5 5 7)_A_($\bar{7}$ $\bar{7}$ 1)_B_ GB without any plastic activity, and the boundary is ultimately separated during the unloading phase in the third cycle. This transformation can be explained by the fact that cyclic loading encourages H accumulation around the crack tip along the boundary. As seen in the inserts of Figure 8a,c, cyclic loading concentrates the stress field ahead of the crack tip, which provides a driving force for the H accumulation. During several cycles, the H atoms in the bulk diffuse into the GB region ahead of the crack tip, as evidenced in Figure 8b,d and Figure 7, where the volume density of segregated H atoms into the GB region is prominently increased, while the volume density in the grain interior is gradually reduced. With the increasing H concentration on the GB, the GB fracture energy is markedly reduced (Figure 2e), thereby favoring the brittle cleavage. Additionally, cyclic loading can aid the crack tip in overcoming the trapping; if a crack propagating along one direction is arrested by a high-disorder region, it alternatively extends through a lower-disorder region along another direction with subsequent cycles (compare the cracking path in insets of Figure 8a,c), thus reducing crack trapping and promoting the cleavage process.

## 4. Discussion and Summary

The simulations show that H segregation creates no ultimate cleavage for the predicted ductile cracks along the studied GBs in bicrystalline α-Fe nanowires under monotonic loading, whereas a ductile-to-brittle transition occurs under cyclic loading. This can be ascribed to the fact that cyclic loading helps accumulate H atoms around the crack tip along the GB and overcome crack trapping in the GB.

Previous studies directly calculated the reduction of GB cohesive properties with varying H concentrations and GB types [58], reporting that at equilibrium concentrations for which embrittlement has been observed in the experiments, the reduction due to H segregation is potentially insufficient to cause an intergranular failure. By considering the inherent competition relationship between brittle cleavage and ductile emission at a crack tip, our results suggest that Fe GBs cannot be simply embrittled by the equilibrium of H segregation into GBs, in accordance with those calculations. Therefore, embrittlement is presumably associated with the H transport process. During cyclic loading, the tensile stress field is concentrated around the crack tip. As the movement velocity of H atoms towards the crack tip is related to the hydrostatic stress, $V\propto \nabla \sigma^{hyd}$ [28], the concentrated stress results in a high H movement. In addition, after several cycles, H atoms in the bulk have ample time to diffuse into the GB region ahead of the crack tip. Under this scenario, ultimate failure is expected to occur.

Aside from H segregation, it is found that crack trapping can also affect the ductile-to-brittle transition. In the case of Σ17 (2 2 3) GB, brittle cleavage is predicted to occur along the [$3\;3\;\bar{4}$] direction with 180 mppm H atoms according to the Griffith’s theory. However, atomistic simulations show that the ductile emission remains the ultimate mechanism. This discrepancy arises mainly due to crack trapping in GBs. Unlike single crystals, GBs typically have complicated atomic structures, the crack tip can be therefore arrested by high-disorder regions of the GB. This blunts the crack tip while encouraging the occurrence of local plasticity. Under cyclic loading, the crack advances dynamically. It is less likely that the crack tip stays dormant and trapped by the GB. In other words, if the crack tip is trapped by a high-disorder region at the loading phase, it may change its position and propagating path at the unloading phase. In this regard, the dynamic propagation alleviates crack trapping and facilitates the cleavage process.

In summary, to elucidate the HE mechanism, the H-modified behavior of intergranular cracks in bicrystalline α-Fe nanowires has been studied using MD simulations. It is found that the twinning emission from the crack tip is favored in the intrinsically ductile directions, and H segregation creates no ultimate cleavage. However, the presence of H atoms causes a significant reduction in the critical stress intensity factor for cleavage and facilitates brittle fracture in the theoretically brittle directions. Furthermore, the simulations show that cyclic loading accumulates H atoms around the crack tip along the boundary and overcomes crack trapping in the GB, and thus induces a ductile-to-brittle transition. These findings enrich our knowledge on experimental observations of H-assisted brittle cleavage failure, and suggest suitable directions for GB engineering of HE-resistant nanostructures.

## Data Availability

The data presented in this study are available on request from the corresponding author.

## Supplementary Materials

The following are available online at https://www.mdpi.com/2079-4991/11/2/294/s1. Video S1: Crack propagation in Σ3 (1 1 2) GB along [$1\;1\;\bar{1}$] direction without H; Video S2: Crack propagation in Σ3 (1 1 2) GB along [$1\;1\;\bar{1}$] direction with 180 mppm H; Video S3: Crack propagation in Σ3 (1 1 2) GB along [$\bar{1}\;\bar{1}\;1$] direction without H; Video S4: Crack propagation in Σ3 (1 1 2) GB along [$\bar{1}\;\bar{1}\;1$] direction with 180 mppm H; Video S5: Crack propagation in Σ5 (2 $\bar{1}$ 0) GB along [$\bar{1}\;\bar{2}\;0$] direction without H; Video S6: Crack propagation in Σ5 (2 $\bar{1}$ 0) GB along [$\bar{1}\;\bar{2}\;0$] direction with 180 mppm H; Video S7: Crack propagation in Σ5 (2 $\bar{1}$ 0) GB along [1 2 0] direction without H; Video S8: Crack propagation in Σ5 (2 $\bar{1}$ 0) GB along [1 2 0] direction with 180 mppm H.
